# Supplementary material for: Predicting hypotension, syncope, and fracture risk in patients indicated for antihypertensive treatment: the STRATIFY models
Source: Nat Commun. 2025 Oct 23;16:9371. doi: 10.1038/s41467-025-64408-9 (PMC12550095; doi:10.1038/s41467-025-64408-9)
Supplement: Supplementary file 2 — Reporting Summary [file 41467_2025_64408_MOESM2_ESM.pdf]

nature portfolio

Corresponding author(s): Constantinos Koshiaris

Last updated by author(s): May 29, 2025

Reporting Summary

Nature Portfolio wishes to improve the reproducibility of the work that we publish. This form provides structure for consistency and transparency in reporting. For further information on Nature Portfolio policies, see our Editorial Policies and the Editorial Policy Checklist.

Statistics

For all statistical analyses, confirm that the following items are present in the figure legend, table legend, main text, or Methods section.

n/a	Confirmed	
☐	☒	The exact sample size (*n*) for each experimental group/condition, given as a discrete number and unit of measurement
☐	☒	A statement on whether measurements were taken from distinct samples or whether the same sample was measured repeatedly
☐	☒	The statistical test(s) used AND whether they are one- or two-sided<br>*Only common tests should be described solely by name; describe more complex techniques in the Methods section.*
☐	☒	A description of all covariates tested
☐	☒	A description of any assumptions or corrections, such as tests of normality and adjustment for multiple comparisons
☐	☒	A full description of the statistical parameters including central tendency (e.g. means) or other basic estimates (e.g. regression coefficient) AND variation (e.g. standard deviation) or associated estimates of uncertainty (e.g. confidence intervals)
☐	☒	For null hypothesis testing, the test statistic (e.g. *F*, *t*, *r*) with confidence intervals, effect sizes, degrees of freedom and *P* value noted<br>*Give P values as exact values whenever suitable.*
☒	☐	For Bayesian analysis, information on the choice of priors and Markov chain Monte Carlo settings
☒	☐	For hierarchical and complex designs, identification of the appropriate level for tests and full reporting of outcomes
☐	☒	Estimates of effect sizes (e.g. Cohen's *d*, Pearson's *r*), indicating how they were calculated

Our web collection on statistics for biologists contains articles on many of the points above.

Software and code

Policy information about availability of computer code

Data collection	No software was used for data collection. Data were provided by CPRD (Clinical Practice Research Datalink) upon protocol approval. Data management was conducted using STATA 16
Data analysis	The analysis was conducted using the statistical software R versions 4.02 and 4.1.1 and STATA 16. Primary package used for the analysis in R was fastcmprsk. Further packages included mice, dplyr, flextable, foreign, tidyr, matrixStats, mfp and visreg.

For manuscripts utilizing custom algorithms or software that are central to the research but not yet described in published literature, software must be made available to editors and reviewers. We strongly encourage code deposition in a community repository (e.g. GitHub). See the Nature Portfolio guidelines for submitting code & software for further information.

Data

Policy information about availability of data

All manuscripts must include a data availability statement. This statement should provide the following information, where applicable:
- Accession codes, unique identifiers, or web links for publicly available datasets
- A description of any restrictions on data availability
- For clinical datasets or third party data, please ensure that the statement adheres to our policy

Data were obtained via a CPRD institutional license. Requests for data sharing should be made directly to the CPRD.

The developed algorithms are freely available for research use and can be downloaded fromhttps://process.innovation.ox.ac.uk/software/. Code lists used to define variables included in the dataset are available at https://github.com/jamessheppard48/STRATIFY-BP (DOI: 10.5281/zenodo.15481343).

Research involving human participants, their data, or biological material

Policy information about studies with human participants or human data. See also policy information about sex, gender (identity/presentation), and sexual orientation and race, ethnicity and racism.

Reporting on sex and gender	Sex was included as a predictor variable in the clinical prediction models developed for this study, allowing for separate predictions for both sexes. Additionally, the performance of the models was evaluated overall as well as within distinct subgroups, including sex, to assess whether there were any differences in predictive accuracy between male and female participants. Data on sex were extracted from the electronic health records (EHR) of GP practices in the UK
Reporting on race, ethnicity, or other socially relevant groupings	Ethnicity was coded as White, Black, South Asian, and Other, and was used as a predictor variable in the clinical prediction models. Ethnicity data were extracted from the electronic health records (EHR) of GP practices in the UK and the Hospital Episode Statistics data (HES) . The performance of the models was assessed both overall and within distinct ethnic subgroups to evaluate whether there were any differences in predictive accuracy across the various ethnic categories. In addition the IMD (Index of Multiple Deprivation) was used as a predictor in all models.
Population characteristics	The study included a range of characteristics, such as demographic factors (age, sex, ethnicity, socioeconomic status, BMI, smoking, alcohol consumption), comorbidities, and medications used by participants (e.g., antihypertensive drugs)
Recruitment	This was a longitudinal cohort study using retrospective data from Electronic Health Care records from the CPRD database (Clinical Practice Research Datalink). Eligible patients had to be registered at a linked general practice in England that contributed data to the CPRD between 1st January 1998 and 31st December 2018. Patients were eligible for analysis if they were 40 years of age or older, registered at a CPRD 'up-to-standard' practice, had available records during the study period, and had a systolic blood pressure reading of 130 mmHg or higher
Ethics oversight	The study protocol was approved by CPRD's Independent Scientific Advisory Committee in February 2019 before obtaining the data relevant to the project (protocol given in the eAppendix in the Supplement). All data are fully anonymised so consent was not required. A project summary is published on the CPRD website (https://www.cprd.com/isac).

Note that full information on the approval of the study protocol must also be provided in the manuscript.

Field-specific reporting

Please select the one below that is the best fit for your research. If you are not sure, read the appropriate sections before making your selection.

☐ Life sciences ☒ Behavioural & social sciences ☐ Ecological, evolutionary & environmental sciences

For a reference copy of the document with all sections, see nature.com/documents/nr-reporting-summary-flat.pdf

Behavioural & social sciences study design

All studies must disclose on these points even when the disclosure is negative.

Study description	Quantitative study using a longitudinal cohort design using retrospective data from Electronic Health Care records from the CPRD database (Clinical Practice Research Datalink) that aimed to develop and externally validate three new prediction models for adverse events commonly associated with antihypertensive treatment—namely hypotension, syncope, and fracture
Research sample	The study was conducted using data from the CPRD GOLD which included primary care data from general practice surgeries that use Vision electronic health record software (Cegedim Healthcare Solutions, London, England) and CPRD AURUM which use data from GP practices using recording software from Egton Medical Information Systems (EMIS, Leeds, England). These data were linked to Office for National Statistics (ONS) mortality data, Hospital Episode Statistics (HES) and Index of Multiple Deprivation data (IMD). The CPRD is generally representative of the UK primary care population.<br>Eligible patients had to be registered at a linked general practice in England that contributed data to the CPRD between 1st January 1998 and 31st December 2018. Patients were eligible for analysis if they were aged 40 years or older, registered at a CPRD 'up-to-standard' practice, had available records during the study period, and had a systolic blood pressure reading of 130 mmHg or higher. Patients with a systolic blood pressure reading ≥180 mm Hg were excluded from the cohort.
Sampling strategy	All patients who met the inclusion criteria were included in the analysis. Outcome and predictor variables were defined using READ codes and ICD-10 codes compiled by the research team. The code lists used to define the variables included in the dataset are available at https://github.com/jamessheppard48/STRATIFY-BP (DOI: 10.5281/zenodo.15481343).<br><br>The pre-specified sample size calculation for model development resulted in an events per variable (EPV) ranging from 7 (Hypotension model) to 20 (Fracture model), assuming an event rate of between 18 and 51 per 10,000 patient years of follow-up, an expected median follow up of 7 years, an estimate of Nagelkerke's R2 statistic of 0.15 and a maximum number of 40 predictor parameters in the model. Based on the above EPV the number of events required ranged from 277 to 784.

	For the external validation, the estimated sample size required for syncope was 8000 patients with at least 400 experiencing the outcome which was sufficient to target a 95% confidence interval (CI) of width 0.2 around the estimate of the calibration slope. This was based on the following assumptions: a skew normal distribution for the model's linear predictor with a mean of 0.16, a variance of 0.5, a skewness parameter of 1, and a kurtosis parameter of 4; an assumed exponential distribution of survival times, with baseline rate parameter 0.008 to ensure 89% survival at 10 years; and a constant censoring rate, with censoring times following an exponential distribution with a rate parameter of 0.2 (to a give a probability of censoring by 10 years of about 87%). Similar numbers were estimated for the other outcomes. The sample sizes in both derivation and validation datasets far exceeded the estimated sample sizes.
Data collection	Data for this study were extracted from electronic health care records in primary care settings across the UK. These records were provided by the Clinical Practice Research Datalink (CPRD) after protocol approval.
Timing	The study period was from 1st January 1998 and 31st December 2018
Data exclusions	No additional data were excluded from the analysis beyond those specified in the exclusion criteria. Missing data at the analysis stage were handled using multiple imputation.
Non-participation	Participants who were lost to follow-up (i.e. transferring out of practice) were censored at that specific time point, and their data were included in the analysis up until that point
Randomization	No randomization took place as this was an observational study

Reporting for specific materials, systems and methods

We require information from authors about some types of materials, experimental systems and methods used in many studies. Here, indicate whether each material, system or method listed is relevant to your study. If you are not sure if a list item applies to your research, read the appropriate section before selecting a response.

Materials & experimental systems

n/a	Involved in the study
☒	☐ Antibodies
☒	☐ Eukaryotic cell lines
☒	☐ Palaeontology and archaeology
☒	☐ Animals and other organisms
☐	☒ Clinical data
☒	☐ Dual use research of concern
☒	☐ Plants

Methods

n/a	Involved in the study
☒	☐ ChIP-seq
☒	☐ Flow cytometry
☒	☐ MRI-based neuroimaging

Clinical data

Policy information about clinical studies

All manuscripts should comply with the ICMJEguidelines for publication of clinical research and a completedCONSORT checklist must be included with all submissions.

Clinical trial registration	This study was not a clinical trial but a Longitudinal cohort study using retrospective data from Electronic Health Care records
Study protocol	The study protocol was approved by CPRD's Independent Scientific Advisory Committee in February 2019 before obtaining the data relevant to the project
Data collection	Data from Electronic Health Records, specifically from the Clinical Practice Research Datalink (CPRD)
Outcomes	Hospitalization or death due to syncope, hypotension, and fracture (distinct outcomes)

Plants

Seed stocks	NA
Novel plant genotypes	NA
Authentication	NA
